# Ovarian teratoma in a teenager causing ureterohydronephrosis: Case report and literature review

**DOI:** 10.1097/MD.0000000000026472

**Published:** 2021-07-09

**Authors:** Dana-Teodora Anton-Păduraru, Ingrith Crenguta Miron, Vasile Valeriu Lupu, Ancuta Lupu, Elena Hanganu

**Affiliations:** a Discipline of Pediatrics; b Discipline of Rehabilitation in Pediatrics, University of Medicine and Pharmacy “Gr. T. Popa”, Iasi, Romania.

**Keywords:** laparotomy, ovary, teenager, teratoma, torsion

## Abstract

**Rationale:**

Teratomas are solid tumors that may occur in both gonadal and extragonadal locations, depending on the age of the child. Benign cystic teratomas are relatively common tumors among women of reproductive age, but they can occur at any age. The clinical presentation is not specific. They can be found incidentally when patients are investigated for other conditions or they can present as emergencies when the ovarian teratoma is torsioned or ruptured.

**Patient concerns:**

We present the case of a 17-year-old adolescent girl that was seen in our emergency department on several occasions for recurrent episodes of abdominal pain ongoing for 6 months.

**Diagnosis:**

An ultrasonography (US) was performed as an outpatient and a left ovarian mass was found along with right ureterohydronephrosis (UHN). Further assessment of the mass was done by abdominal and pelvic CT and tumoral markers. CT appearance was more suggestive of a teratoma.

**Interventions:**

She underwent laparotomy with complete excision of the tumor.

**Outcome:**

The patient had an uneventful recovery. A renal US follow up showed reduction of the dilatation, demonstrating that the condition was secondary to tumor compression.

**Lessons:**

In a teenager with nonspecific symptoms, a high suspicion index for tumors is mandatory. An early diagnosis and management avoid complications like UHN.

## 1 Introduction

Teratomas (from the Greek word *terato* - “a monster” and *onkoma* - “mass or swelling”) are embryonal neoplasms consisting of tissues from 2 or 3 germ layers. Ovarian tumors in children have different characteristics regarding incidence, histology, and clinical presentation compared to those in adult population.^[1]^ The embryological basis of teratomas starts with the formation of germ layers such as ectoderm, endoderm, and mesoderm. According to the WHO classification, the group of germ cell tumors includes mature, immature teratomas, and teratomas with potential of malignant transformation.^[2]^ The classification of Gonzales-Crussi from 1982 identifies mature teratomas (G0) as more frequent (54.5%) and immature teratomas less frequent (45.5%).^[2,3]^ Ovarian teratomas are the most common germ tumors that are characterized by the presence of mature and immature structures. The most frequent teratoma in children is the mature cystic type also known as dermoid cyst.^[4]^ Germ-cell tumors show a distinct genetic profile. Oosterhuis et al grouped the ovarian tumors on the basis of their chromosomal abnormalities, and opined that mature cystic teratomas occur due to numeric abnormalities such as extra X, 7, 12, and 15. Contrarily, Cushing et al^[5]^ reported that 95% of teratomas are karyotypically normal.^[5]^ Hara et al, cited by Stanton,^[6]^ concluded that one of the mechanism incriminated in the pathogenesis of teratomas can be related to the *MAGE* gene family of tumor rejection antigens.^[6]^

## 2 Case report

A 17-year-old girl was presented to emergency department (ED) with abdominal pain ongoing for the last 5 days, associated with nausea and headache. The pain was localized in the lower abdomen, described as sharp and stabbing in nature, intermittently and was not radiating. Her periods were normal and no other associated symptoms. She has had 4 previous presentations to ED for similar episodes. The pain was not as severe as at the current presentation and she was sent home on oral analgesia. Her family history revealed some affective deficiencies but no other significant background.

On examination, she was in good general state, normal vitals, afebrile, with a body mass index of 22.79 kg/m^2^. Her abdomen had generalized tenderness, not guarding, and there was no palpable mass.

The results of a complete blood count and serum biochemistry were normal and a urine pregnancy test was negative.

**Figure 1:**
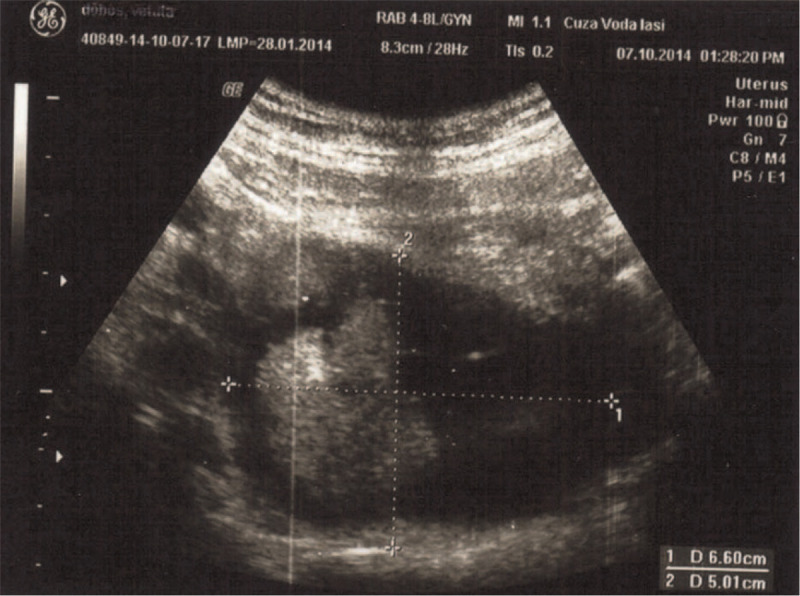
Ovarian teratoma-ultrasound image: parauterine teratoma, left ovary with a normal structure.

An abdominal ultrasound was performed on this occasion and the presence of mass was demonstrated in the right para uterine region. The US dimensions were size 137 × 72 × 57 mm with a heterogeneous echo structure (solid and liquid), and a calcification of 7 mm on the posterolateral side of the tumor was also described. Solid structures seemed avascular. The right ovary was not identifiable as a separate entity, possibly embedded in the tumor and the left ovary was 33 × 20 mm with a normal structure on echography. There was no evidence of ovarian torsion. The uterus was regular in shape and size, 42 × 37 × 65 mm, Figure 1. The right kidney was hypoplastic, 74 × 30 mm, with evidence of grade III ureterohydronephrosis on the left side.

Further, a computed tomography (CT) was performed and this revealed an expansive heterogeneous tumor 97 × 129 × 148 mm, suggestive of an ovarian teratoma with a Rokitansky nodule of 50 × 47 × 61 mm on the posterolateral wall.

Tumoral markers---alpha fetoprotein (AFP) level and β-human chorionic gonadotrophin (β-HCG)---were normal.

**Figure 2:**
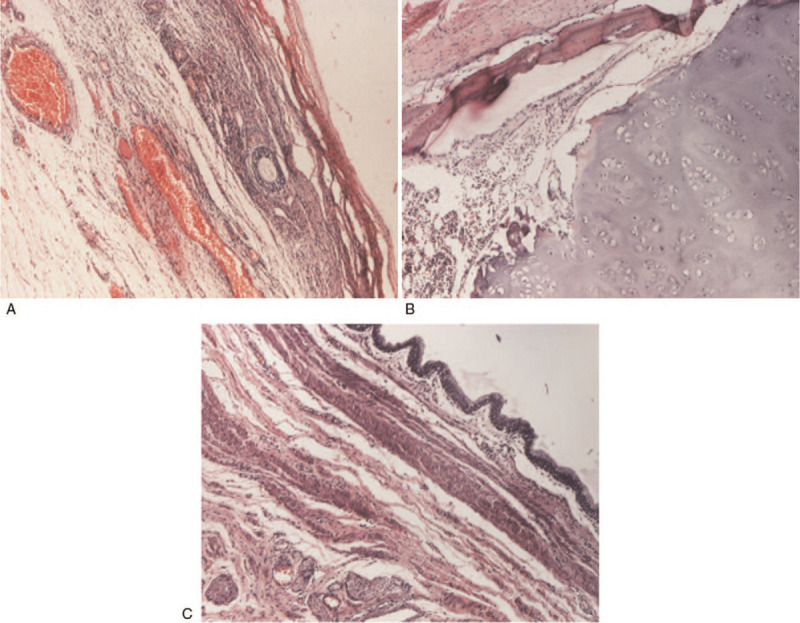
Ovarian teratoma: histopathologycal report with mature structures. (A) Ovarian parenchyma (HEx40). (B) Hyaline cartilage, bone marrow, bone blades (HEx40). (C) Cystic formation dusted by mucous-secreting epithelium and smooth muscle wall (HE x100).

Decision for tumor excision was made and the patient was scheduled for surgery the following week. A laparotomy through a Phannenstiel incision was performed. The right ovary was occupied entirely by the large mass, so a complete excision of the right ovary along with the tumor was performed. Histopathology report described mature structures such as squamous epithelium, respiratory epithelium, bone, cartilage, bone marrow, nervous tissue, lymph nodes, smooth muscle, fat and glandular tissue, lymphoid tissue, and gastric antrum, Figure 2 (A---C) consisting with a solid ovarian teratoma.

Postoperative recovery period recorded no complication and the patient was discharged after 5 days. Short-term follow-up showed an excellent outcome with no abdominal pain and good cosmetic appearance of the scar. Interestingly, the patient also reported the resolution of her long-standing symptoms of headaches. Short-term follow-up, which included abdominal and renal ultrasound, showed the resolution of ureterohydronephrosis from grade III on the left side to normal.

## 3 Discussion

Ovarian masses are uncommon findings in adolescent girls.^[7–12]^ The presence of gonadal tumors increases with the onset of puberty and peaks around 15 to 19 years of age.

A literature search was conducted using PubMed publications to search case reports, literature reviews, and case series published from January 1970 through January 2019 with the following keywords ovarian teratoma, teenager, hydronephrosis.

We excluded reports available only as abstracts or published in other language than English or Romanian. The literature search identified 1292 results from which 182 full text articles in English were selected. From this, only 20 were free access studies with 5 of them concerning more than 50 patients. These 5 studies were included in our review. Scientific articles were selected and reviewed to assess the incidence, clinical presentation, diagnosis, treatment, and anatomopathological characteristics of ovarian teratomas in teenagers.

Ovarian germ cell tumors account for about 30% of germ cell tumors and 70% of all neoplastic ovarian masses, being the most common ovarian neoplasms in children and teenagers. Benign and immature teratomas constitute about 80% of all ovarian germ cell tumors and are bilateral in 5% of cases, whereas the incidence of malignant forms is reported in about 20% and increases during adolescence.^[13]^ Fifty-seven percent of patients were diagnosed between 9 and 15 years of age. Mature teratoma was diagnosed in 83% patients, while 17% patients presented with immature teratoma. Presentation of patients with ovarian teratomas is similar in different group age; abdominal pain (70–80%) and lower abdominal mass are the most common symptoms. The tumor is often asymptomatic until it reaches a very large size with compression of the neighboring structures, including the retroperitoneal ureter causing ureterohydronephrosis similar to our patient.^[14]^ About 10% of cases present as acute abdomen due to torsion, infarction, or spontaneous rupture of the mass. Evaluation of serum markers is essential to address the nature of the tumor. Increased αFP or βHCG indicates a malignancy αFP is raised in over 90% of children with malignant teratomas while βHCG is elevated in 30% of cases malignant cases.^[13]^

The only effective therapy for ovarian teratomas is complete surgical removal. Sixty-five percent of girls with mature teratoma had laparotomy and 35% had laparoscopy performed as an initial operative approach.^[15]^ The use of laparoscopy to remove highly suspected malignant lesions is usually discouraged due to the size of the mass and its invasiveness.^[13]^ This is the reason for open surgical treatment and not minimal invasive in our case.

According to Stanton,^[6]^ gonadal tumors were encountered in less than 10% of the cases in the 0 to 5-year-old age group, 20% in the 5 to 9-year-old age group and the vast majority of 70% were reported in the 10 to 14-year-old age group. Germ cell tumors account for 55% of the ovarian tumors in the adolescent population.^[10]^ From the group of ovarian tumors, mature cystic teratomas represent a segment of 10% to 20% and usually these are with benign histology but in less than 1% malignant transformation is reported.^[16]^

According to our experience in female patients, there is a higher incidence of germ cell tumors but a higher suspicion of malignant tumors in male patients. No racial predispositions for these tumors are known yet.

The site of origin of teratomas is related to patient age group. For infants and preschool children, there is a higher incidence of extragonadal teratomas, and in older children and adolescents, there is a higher incidence of gonadal teratomas. Among the ovarian tumors, dysgerminoma is the most prevalent during puberty.^[17,18]^ In about 10% of the cases, these tumors are bilateral.^[5]^

Teratomas can be explained through the theory of abnormal differentiation of fetal germ cells that originate from the fetal yolk sac. According to this theory, a process of normal migration of the germ cells is related to the development of gonadal tumors, whereas a process of abnormal migration is correlated to the development of extragonadal tumors.^[6]^

Another theory suggests that ovarian germ cell tumors in general and teratomas in particular may develop directly from oocytes. In fact, one of the most incriminated theories for the development of ovarian teratomas is the theory of parthenogenesis’ activation of oocytes.^[16]^

Diets high in polyunsaturated fat represent a risk factor in the development of teratomas. It has been demonstrated that plant estrogens are possibly associated with an increased tumor risk.^[6]^ Patients with ovarian tumors usually have a modified hormonal activity and/or even clinical signs of abnormal sexual development.^[1]^

According to their content, the teratomas are classified as:

(1) solid teratoma (contains only tissues);
(2) cystic teratoma (contains fluid: fat, cerebrospinal fluid, or sebum);
(3) mixed teratoma (contains cystic and solid parts).^[2,5]^

Typically, the mature cystic teratoma contains mature tissues of different origins: endodermal (bronchial epithelium, thyroid tissue, gastrointestinal epithelium), mesodermal (cartilage, bone, muscle, fat), and ectodermal (skin, brain).^[5,19]^ Mesodermal tissue is present in 90% of cases, in 67% to 75% of cases there is adipose tissue, and in 31% of cases teeth. They have a slow rate of growth, in average 1.8 mm/year. In 75% of the cases, their diameter is less than 10 cm and seldom more than 15 to 25 cm.^[10,20,21]^ In about 10% to 15% of the cases, these tumors are bilateral.^[4,20]^

Some mature cystic teratomas are asymptomatic, silent (13–66%), and are discovered incidentally through imaging studies.^[10,21,22]^ Still, ovarian tumors can be detected through physical examination as a palpable mass or can present as an emergency. The growth of an ovarian tumor typically produces pain in the lower abdomen (primary symptom), abdominal distension, and/or emesis. Acute and chronic pain can occur with equal frequency. Abdominal pain is caused by torsion of the ovarian mass or irritation of the ovarian ligaments. Ovarian tumors that are palpable are less encountered and appear later in the evolution. Larger tumors are more likely to be diagnosed early.^[6]^

Germ cell tumors have different possibilities of histologic differentiation, still the histological pattern is similar regardless of their primary site of origin. The characteristic pathologic appearance is squamous epithelium lining the wall of the cyst and hyalinized ovarian stroma often covering the external surface. The tumors contain sebaceous material which is liquid at body temperature or semisolid at room temperature. Hair, muscle, skin glands, and other tissues lie within the wall. Microscopically, skin elements dominate, including dermal appendages such as hair follicles and sebaceous glands. Usually, a protuberance named a Rokitansky nodule projects into the cyst cavity. If bone or teeth are present, they are found within this nodule. Invariably, the ectodermal tissue (neural tissue, skin derivatives) exists. In 90% of the cases, there is mesodermal tissue (fat, bone, cartilage, muscle) and rare endodermal tissue (bronchial, thyroid, and gastrointestinal epithelium). The rarer ovarian teratomas that contain thyroid tissue are called struma ovarii. Teeth are seen in 31% of cases and adipose tissue in 65% to 75% of cases.^[4,19]^ Benign cystic teratomas have a low malignant potential, which increases in direct proportion to the solid component of the tumor.^[21]^

Occasionally, the components of mature teratoma are biologically active, secreting hormones (insulin, prolactin, vasopressin, growth hormone) and enzymes.^[5]^

The diagnosis is complicated because mature teratomas have different presentations:

(1) a cystic lesion with a densely echogenic tubercle projecting into the cyst lumen (Rokitansky nodule);
(2) a mass with the echogenic area with sound attenuation due to the sebaceous material and hair within the cyst cavity;
(3) many echogenic bands, thin due to the hair in the cyst cavity.^[4]^

The 2 main markers produced by germ cell tumors are AFP and β-HCG. AFP and/or β-HCG help in establishing the clinical diagnosis of tumors presenting at a typical localization. Detection of AFP secreted by yolk sac elements is also used as a marker for treatment efficacy or recurrence.^[23]^ Girls younger than 15 years who have large, solid teratomas on ultrasonography or positive markers for germ cell tumors are at risk for malignant variants.^[24]^ Still, patients with negative tumor markers are not excluded from the risk group of malignancy.^[1]^

Diagnostic imaging is an essential part of initial staging. Ultrasonography is the most commonly used diagnostic tool for tumors. When 2 or more characteristic findings for mature tumors, such as shadowing echo density and regionally bright echo density are present, the positive predictive ability of ultrasonography approaches 100%.^[25,26]^ Ultrasonography of the pelvis and abdomen is an extremely useful examination for the measurement of the spread of the ovarian tumor without the risk of ionizing radiation.^[6,27]^ Ultrasonography is used to define the size of the lesion and to characterize its gross morphologic condition as solid, simple cyst, or complex cyst.^[10,28]^ CT examination of the pelvis and abdomen is considered mandatory for correct staging of any pelvic tumor at the time of diagnosis. In selected cases, an MRI of the pelvis and abdomen can be performed instead of CT scan.^[6]^

The differential diagnosis of the ovarian tumors in pediatric population will be based on the particularities of clinical manifestation with abnormal levels of serum tumor markers and the pathognomonic imagistic characteristics.^[1]^

Mature cystic teratomas can be associated with complications: torsion (3–16%; the most common complication), rupture associated with peritonitis (1–4%), malignant degeneration (1–2% of the cases), superimposed infection (1%), and autoimmune hemolytic anemia (<1%).^[10,20]^

The treatment of choice for ovarian teratomas is surgical removal. For the development of normal puberty and future fertility, it is important to perform conservative ovarian surgery in childhood and adolescence.^[1,11,15,22,25,29,30]^ The importance of cosmetic appearance to the psychological well-being of a young adolescent woman should not be overemphasized.^[21]^ Surgical intervention should aim toward preservation of ovarian tissue.^[7]^ In our case, the result of using Pfannenstiel incision was an improved cosmetic outcome without compromising the patient's safety and a shorter hospitalization period.

The risk of recurrence depends on the primary site, on the histological grade of immaturity, and completeness of the primary resection.^[14]^ In young patients with mature tumors (bilateral or multiple), there is a 2% to 3% incidence of the subsequent development of germ cell tumors.^[25]^

There are studies that reported cases of cystic teratoma involving headache. In a case, teratoma included the presence of neural tissue, like in our patient.^[31]^ That case also had behavioral changes that were not seen in our teenager. Also, another case of teratoma involving headache described that patient was without behavioral changes and without neural tissue in the tumor.^[32]^ Also, there is a reported case of acute urinary retention caused by a mature cystic ovarian teratoma.^[33]^

The follow-up includes physical examination, observation, measurement of AFP and/or β-HCG, and scanning (ultrasonography, CT, MRI).^[2]^

## 4 Conclusion

For an ovarian mass, there is no specific symptom or physical examination finding characteristic. Because of the nonspecific nature of presenting symptoms, even in the instance of ovarian mass torsion, the diagnosis can be delayed, especially in cases where spontaneous detorsion occurs. As many of the presenting symptoms seen in ovarian teratomas overlap with other diseases, imaging is a very important part of the diagnostic workup. In a teenager with nonspecific symptoms, a high suspicion index for tumors is mandatory. An early diagnosis and management avoid complications such as UHN.

## Acknowledgments

The authors would like to thank to the Radiology Department, Pathology Department, Dr. Petru Plămădeală and Dr. Doina Mihăilă for their contribution in managing this patient.

All authors contributed equally to this paper.

## Author contributions

**Conceptualization:** Dana-Teodora Anton-Paduraru, Ingrith Crenguta Miron, Vasile Valeriu Lupu, Ancuta Lupu, Elena Hanganu.

**Investigation:** Dana-Teodora Anton-Paduraru, Ingrith Crenguta Miron, Vasile Valeriu Lupu, Ancuta Lupu, Elena Hanganu.

**Validation:** Dana-Teodora Anton-Paduraru, Ingrith Crenguta Miron, Vasile Valeriu Lupu, Ancuta Lupu, Elena Hanganu.

**Writing – original draft:** Dana-Teodora Anton-Paduraru, Ingrith Crenguta Miron, Vasile Valeriu Lupu, Ancuta Lupu, Elena Hanganu.

**Writing – review & editing:** Dana-Teodora Anton-Paduraru, Ingrith Crenguta Miron, Vasile Valeriu Lupu, Ancuta Lupu, Elena Hanganu.
